# Effect of Secretion Efficiency of Mutant KRAS Neoantigen by *Lactococcus lactis* on the Immune Response of a Mucosal Vaccine Delivery Vehicle Targeting Colorectal Cancer

**DOI:** 10.3390/ijms24108928

**Published:** 2023-05-18

**Authors:** Nur Aqlili Riana Alias, Winfrey Pui Yee Hoo, Pui Yan Siak, Siti Sarah Othman, Noorjahan Banu Mohammed Alitheen, Lionel Lian Aun In, Raha Abdul Rahim, Adelene Ai-Lian Song

**Affiliations:** 1 Department of Cell and Molecular Biology, Faculty of Biotechnology and Biomolecular Sciences, Universiti Putra Malaysia, Serdang 43400, Malaysia; aqliliriana.alias@gmail.com (N.A.R.A.); sarahothman@upm.edu.my (S.S.O.); noorjahan@upm.edu.my (N.B.M.A.); raha@upm.edu.my (R.A.R.); 2 Department of Biochemistry and Molecular Biology, Monash Biomedicine Discovery Institute, Monash University, Clayton, VIC 3800, Australia; winfrey.hoo@monash.edu; 3 Faculty of Medicine and Health Sciences, UCSI University, Bandar Springhill, Port Dickson 71010, Malaysia; siakpy@ucsiuniversity.edu.my; 4 UPM-MAKNA Cancer Research Laboratory, Institute of Bioscience, Universiti Putra Malaysia, Serdang 43400, Malaysia; 5 Department of Biotechnology, Faculty of Applied Sciences, UCSI University Kuala Lumpur, Cheras 56000, Malaysia; lionelin@ucsiuniversity.edu.my; 6 National Institutes of Biotechnology Malaysia, Argo-Biotechnology Institute Malaysia Complex, Serdang 43400, Malaysia; 7 Department of Microbiology, Faculty of Biotechnology and Biomolecular Sciences, Universiti Putra Malaysia, Serdang 43400, Malaysia; 8 Laboratory of Vaccine and Biomolecules, Institute of Bioscience, Universiti Putra Malaysia, Serdang 43400, Malaysia

**Keywords:** secretion, mutant KRAS antigen, signal peptide, SPK1, mucosal vaccine delivery, *Lactococcus lactis*, immunotherapy, colorectal cancer

## Abstract

Colorectal cancer (CRC) is often caused by mutations in the KRAS oncogene, making KRAS neoantigens a promising vaccine candidate for immunotherapy. Secreting KRAS antigens using live Generally Recognized as Safe (GRAS) vaccine delivery hosts such as *Lactococcus lactis* is deemed to be an effective strategy in inducing specific desired responses. Recently, through the engineering of a novel signal peptide SPK1 from *Pediococcus pentosaceus,* an optimized secretion system was developed in the *L. lactis* NZ9000 host. In this study, the potential of the *L. lactis* NZ9000 as a vaccine delivery host for the production of two KRAS oncopeptides (mutant 68V-DT and wild-type KRAS) through the use of the signal peptide SPK1 and its mutated derivative (SPKM19) was investigated. The expression and secretion efficiency analyses of KRAS peptides from *L. lactis* were performed in vitro and in vivo in BALB/c mice. Contradictory to our previous study using the reporter staphylococcal nuclease (NUC), the yield of secreted KRAS antigens mediated by the target mutant signal peptide SPKM19 was significantly lower (by ~1.3-folds) compared to the wild-type SPK1. Consistently, a superior elevation of IgA response against KRAS aided by SPK1 rather than mutant SPKM19 was observed. Despite the lower specific IgA response for SPKM19, a positive IgA immune response from mice intestinal washes was successfully triggered following immunization. Size and secondary conformation of the mature proteins are suggested to be the contributing factors for these discrepancies. This study proves the potential of *L. lactis* NZ9000 as a host for oral vaccine delivery due to its ability to evoke the desired mucosal immune response in the gastrointestinal tract of mice.

## 1. Introduction

Colorectal cancer (CRC) is the third most commonly diagnosed cancer in both men and women. It accounted for over 1.9 million new cases in 2020 and 935,000 estimated annual deaths, globally, thus possessing the second highest mortality rate after lung cancer (Sung et al., 2021) [1]. CRC occurrence is commonly associated with symptoms including changes in bowel habits, rectal bleeding, blood in the stool, abdominal cramping or pain, weakness, fatigue, and unintended weight loss (Kanthan et al., 2012) [2]. Several risk factors highly associated with CRC include obesity, a diet rich in red meat, smoking, and alcohol consumption, with people above 50 years old and those that have been exposed to inflammatory bowel syndromes or genetic mutation inheritance having higher chances of developing the disease (Brenner et al., 2014) [3].

The Kirsten-ras (*KRAS*) proto-oncogene is one of the most frequently mutated oncogenes in CRCs besides pancreatic ductal adenocarcinomas (PDACs) and non-small cell lung carcinomas (NSCLCs) (Fernández-Medarde and Santos, 2011, McCormick, 2015) [4,5]. *KRAS* is involved in various essential cellular functions, including cell proliferation, apoptosis, migration, and cell differentiation (Arrington et al., 2012) [6]. The significant role of *KRAS* mutations besides other oncogenes such as *BRAF*, *p53*, and *SMAD4* in CRC cancer progression and metastasis has been known to arise early during the progression from colorectal adenoma to malignant carcinoma and has alarmingly affected Asian populations (Huang et al., 2018) [7]. A somatic point mutation in the *KRAS*, which is predominantly found in codon 12 (G12V) and 13 (G13D), accounts for about 35–45% of the advanced metastatic stage of CRC (mCRC) (Fernández-Medarde and Santos, 2011) [4].

*KRAS* gene mutations are often linked with poor response to treatment and increased risk of cancer recurrence with a low survival rate. While the five-year survival rate for patients in earlier stages is approximately 90%, the survival rate decreases significantly to less than 12% for patients in the advanced metastatic stage (stage IV) (Caiazza et al., 2015) [8]. An EGFR-targeted therapy using monoclonal antibodies such as panitumumab and cetuximab is among the FDA-approved treatments available against metastatic CRC cancers (Bignucolo et al., 2017) [9]. However, the treatment efficacy is often compromised by the presence of *KRAS* gene mutations downstream of the EGFR pathway. This renders KRAS (+) patients resistant to the treatment and left with limited alternative options such as chemotherapy and radiotherapy, which have poor specificity and impose potential side effects (Arrington et al., 2012) [6]. Therefore, finding a safer, more efficient, and more specific alternative regimen such as immunotherapy is imperative.

Cancer immunotherapy that utilizes a peptide vaccine arising from epitopes of tumor-associated (TAA) or tumor-specific (TSA) neoantigens such as KRAS has the potential to modulate immune responses in a specific manner (Carbone et al., 2005; Toubaji et al., 2008; Rahma et al., 2014; Pan et al., 2019) [10,11,12,13]. Nevertheless, the peptide vaccine is often perturbed by its small size, making it weakly immunogenic and rendering it incapable of inducing a sufficient immune response. These limitations can be surmounted by modification of the TSA/TAAs through the incorporation of an additional single point mutation (Ng et al., 2018) [14] or by conjugation with a carrier molecule to improve the adjuvanticity and chemical stability for enhanced immune response (Li et al., 2014) [15].

To target the peptide vaccine for mucosal administration, a delivery system using an inert vehicle (e.g., liposomes, and microparticles) or a live bacterial vehicle is usually used to improve the delivery of the target antigen to the desired location (Wells and Mercenier, 2008) [16]. Live attenuated bacteria from pathogenic strains are advantageous as delivery hosts due to their ability to evoke a strong and robust immune response, yet they possess the risk of reverting to wild-type phenotypes and are thus unsuitable to be used in children or immunocompromised people (Wells and Mercenier, 2008) [16]. Alternatively, vaccination using a live Generally Recognized as Safe (GRAS) status bacterium such as *Lactococcus lactis* has recently emerged as a safer alternative for mucosal vaccine delivery (Nouaille et al., 2003; Wyszyńska et al., 2015; Vilander and Dean, 2019) [17,18,19].

*L. lactis*, which is also a Gram-positive lactic acid bacteria (LAB) workhorse for protein production, is a great host for mucosal vaccine delivery due to its GRAS status with a long history of use in the food fermentation industry. The bacterium is also non-invasive and non-colonizing, thereby reducing the potential for tolerance towards the target antigens. For efficient vaccine delivery, localization of the target antigen is important, and *L. lactis* offers a strategy of protein production by secretion, which is thought to be more effective than an intracellular or surface-displayed option as secretion allows direct access of the antigen to the target environment (Song et al., 2017) [20], in this case, the gastrointestinal tract. In view of this, recently, an enhanced secretion system in *L. lactis* was developed through the modification of a novel heterologous signal peptide (SP) SPK1 from *Pediococcus pentosaceus*, as an alternative to the most commonly used lactococcal SP USP45. A newly derived SPK1 mutant signal peptide, denoted SPKM19, was shown to mediate improved secretion efficiency of a reporter nuclease protein (NUC) by about 1.4-fold compared to the original SPK1 (Alias et al., 2022) [21].

Thus, in this study, the two SPs, SPK1 and its derivative SPKM19, were employed for the secretion of two therapeutic KRAS mimotopes, 68V-DT and wild-type KRAS, in developing *L. lactis* as a potential mucosal vaccine against metastatic colorectal cancer. The SPs were fused with propeptide LEISSTCDA (LEISS) to further enhance secretion efficiency (Le Loir et al., 1998) [22] and their ability in secreting the KRAS mimotopes was compared in vitro and in vivo. The 68V, which is a modified G12V KRAS mimotope, harbors an additional single point mutation (V7D) at the MHC groove binding regions flanking the original codon 12 mutations. It was previously predicted to promote high affinities towards MHC-II and -I, as well as B-cell epitopes in silico (Ng et al., 2018) [14]. Here, the 68-V was further fused with diphtheria toxoid (DT) to improve the chemical stability and adjuvanticity. Meanwhile, the wild-type KRAS (wt-KRAS), derived from the human KRAS oncogene expressed in all healthy humans, was used as a control. Thus, the aim of study was to develop a lactococcal vaccine against colorectal cancer and to determine the effect of secretion mediated by different SPs on the efficacy of the vaccine.

## 2. Results

### 2.1. In Silico Analysis of KRAS Fusion Proteins

Prior to experimental settings, in silico analysis was performed on the putative fusion proteins, SPKM19-LEISS-68V-DT, SPK1-LEISS-68V-DT, SPKM19-LEISS-wtKRAS, and SPK1-LEISS-wtKRAS to understand their characteristics. The postulated SP performance (D-score) and signal peptidase 1 (SPase I) cleavage site of the different SP-LEISS-KRAS fusions genes are shown (Table 1). The KRAS proteins (68V-DT and wtKRAS) fused to SPKM19-LEISS produced a higher D-score; thus, it was predicted to give better secretion efficiency (SE) as compared to those KRAS proteins fused to the wild-type SPK1-LEISS, as previously shown in our study using the reporter NUC as a target protein (Alias et al., 2022) [21]. The net charge of the SP region for all four fusion proteins was positive, which is desirable.

The predicted sequence and net charge of the mature protein (MP) region of the different SP-KRAS fusions genes following the respective SPs’ cleavage region are also shown (Table 1). There is a slight variation in the first 10 amino acid sequence of the MP region when they are fused with different SPs, where KRAS fused to SPKM19-LEISS has an Ala residue at position +1, due to modification previously performed on the C-terminal of the SPKM19 by insertion of Ala at position −3 (Alias et al., 2022) [21], as opposed to KRAS fused to SPK1-LEISS, which has a Val at position −3. However, the variations in the first 10 residues of each MP did not affect the overall net charge with all four KRAS fusion proteins retaining a similar net negative charge. The fusion of the propeptide LEISSTCDA (LEISS) between the SP and N-terminal region of the KRAS antigens served not only as an enhancer for protein secretion (Le Loir et al., 2001) [23] but also as an anionic linker to maintain the similar negative charge balance of the first 10 MPs, since, without the LEISS fusion, the first 10 MPs of wtKRAS and 68V-DT would have a net charge of 0, respectively. Maintaining the net positive charge of the SP and the net negative charge protein of at least the first 10 and 18 residues of MP were demonstrated in previous studies to positively affect the secretion (Alias et al., 2022; Choo and Ranganathan, 2008; Kajava et al., 2000) [21,24,25].

The physicochemical property and secondary structure of the different MPs when fused to both SPK1-LEISS and SPKM19-LEISS were identified to understand the characteristics of the different SP-MP fusion proteins (Table 2). There is no striking difference observed between the wtKRAS and 68V-DT in regard to the protein size and the first 10 or 18 amino acids of the mature moiety’s charge balance due to the effect of the LEISSTCDA linker. However, there are prominent differences between both KRAS variations with staphylococcal NUC protein, previously used for fusion with SPK1 and SPKM19 (Alias et al., 2022) [21], in regard to the protein sizes and net charges of the MPs. This is suggested to affect the secondary structure confirmation of the SP-MP fusion proteins. An overall global net positive charge for the SP and a global net negative charge for the MP are desired for prokaryotes (Choo and Ranganathan, 2008) [24]. All KRAS proteins carried a net negative charge on the first 10 or 18 residues and a net negative charge for the GRAVY score, indicating the hydrophilic nature of the MP with a stability index less than 40, an indication of a stable MP.

### 2.2. Lactococcal Recombinant Strains Secreting KRAS Mimotopes

**Figure 1:**
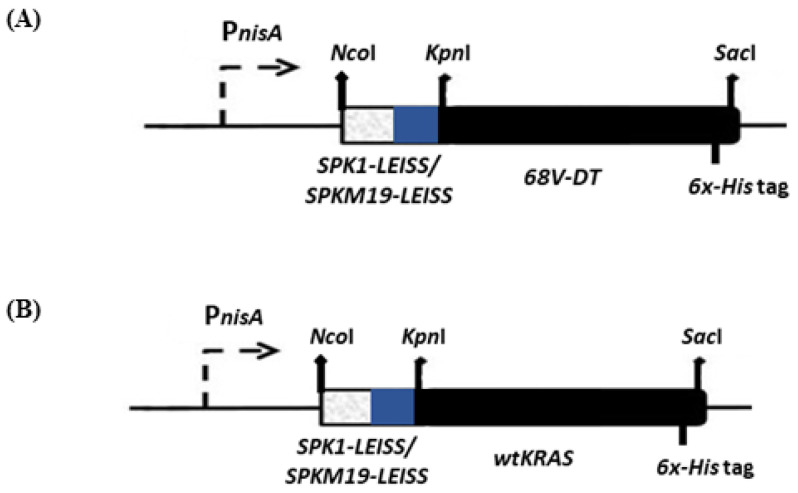
Construction of secretory cassettes of signal peptides and propeptide (*SPK1-LEISS* or *SPKM19-LEISS*) fused to target genes, (**A**) KRAS *68V-DTD* or (**B**) wild-type KRAS (*wt*KRAS). The 9-residue anionic linker, *LEISSTCDA*, was placed immediately downstream of the C-terminal cleavage site of *SPK1* to preserve the net negative charge of the first 10 MP protein as well as an enhancer for secretion. A six-histidine (*6×-His*) tagged sequence was included at the C-terminal ends of target genes for protein detection. The REs (*Nco*I, *Kpn*I, and *Sac*I) used for directional cloning of the genes are shown.

**Figure 2:**
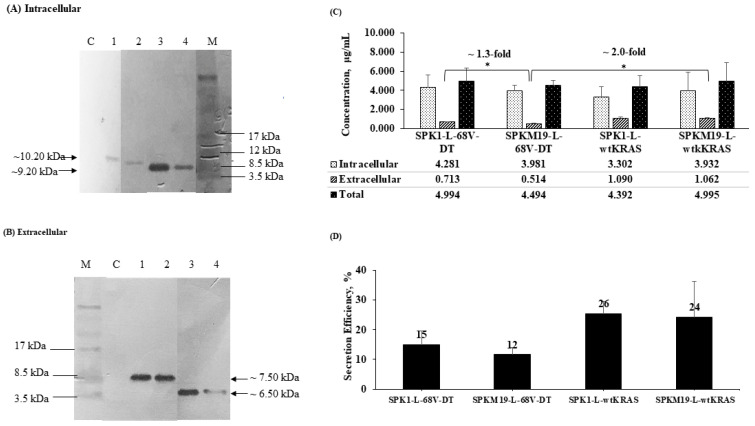
Expression and secretion analysis of KRAS mimotopes produced by recombinants *L. lactis* at 6 h expression with 40 ng/mL of nisin induction. (**A**) Western blotting analysis of KRAS in intracellular fraction extracted from the cell pellet. C: NZ9000 harboring empty vector pNZ8048 (negative control); Lane 1: NZ-SPKM19-L-WT; Lane 2: NZ-SPK1-L-WT; Lane 3: NZ-SPKM19-L-68V-DT; Lane 4: NZ-SPK1-L-68V-DT; M: Rainbow™ Protein Ladder. (**B**) Western blotting analysis of KRAS in the extracellular fraction extracted from the culture supernatant. M: Rainbow™ Protein Ladder; C: NZ9000 harboring empty vector pNZ8048 (negative control); Lane 1: NZ-SPKM19-L-WT; Lane 2: NZ-SPK1-L-WT; Lane 3: NZ-SPKM19-L-68V-DT; Lane 4: NZ-SPK1-L-68V-DT. The unedited Western blots are shown in supplementary data, Scheme S1. (**C**) ELISA analysis of intracellular, secreted, and total yield of KRAS produced by the recombinants *L. lactis.* The significance difference, *p* < 0.05, between different KRAS groups and within KRAS groups was shown as (*). (**D**) Secretion efficiency analysis of KRAS (wtKRAS and 68V-DT) aided by different SPs.

Four different *L. lactis* recombinants were successfully constructed in this study, as shown in Figure 1. The recombinants, which harbored the SPK1-LEISSTCDA or SPKM19-LEISSTCDA fused to target antigens, 68V-DT and wtKRAS, respectively, were denoted as NZ-SPK1-L-68V-DT, NZ-SPKM19-L-68V-DT, NZ-SPK1-L-WT, and NZ-SPKM19-L-WT. As a negative control, an empty plasmid pNZ8048 was also transformed into NZ9000 and denoted as NZ-pNZ8048. The amount of KRAS mimotopes capable of being produced and secreted by the *L. lactis* was determined. As shown from the ELISA analysis (Figure 2), following 6 h expression with 40 ng/mL nisin, all the recombinant *L. lactis* strains successfully expressed and secreted the KRAS proteins. Bands of expected sizes were seen for all strains. The expected sizes for precursors 68V-DT and wtKRAS in the intracellular fractions were ~9.36 kDa and ~10.3 kDa, respectively (Figure 2A). The sizes are inclusive of the SPs (SPK1/SPKM19) and propeptide (LEISS) at the N-terminal ends and the six-Histidinetag (6×-His) at the C-terminal ends of the mimotopes. Meanwhile, the expected sizes of the secreted MPs for 68V-DT and wtKRAS strains in the extracellular fractions were ~6.5 kDa and ~7.5 kDa, respectively (Figure 2B), slightly lower than the intracellular proteins due to cleavage of the SPs by the host SPase I following successful translocation to the extracytoplasmic region.

### 2.3. Yield and Secretion Efficiency of KRAS Mimotopes at 6 h Expression in L. lactis

At 6 h after induction, the yield of secreted KRAS mimotopes produced in the extracellular fractions was ~0.5–1.0 mg/L (Figure 2C). This amount is consistent with the previously reported studies by Li et al. (2015) [26] and Bermúdez-Humarán et al. (2003) [27] for the secretion of IL-6 and IFN-ω proteins using SP USP45 in the *L. lactis* host (~0.2–1.0 mg/L). When comparing the effect of different SPs, the secretion yield of KRAS (68V-DT) aided by the SPK1 was higher than that aided by the SPKM19 (*p* < 0.05). This differs from the earlier in silico prediction based on the D-score value (Table 1) and our previous study using staphylococcal nuclease, NUC, as the target protein (Alias et al., 2022) [21], where a superior SE of the optimized SPKM19 was predicted and observed in comparison with SPK1.

In addition, for all strains, the secreted KRASs were produced at a much lower yield (by approximately 3–7-fold) than the intracellular fraction, indicating inefficient secretion with a large majority of the proteins retained in the cell instead of being translocated out despite the use of SPs. Although an overall low secretion efficiency (SE) was observed as shown in Figure 2D, the total yield of KRAS mimotopes produced by all four *L. lactis* recombinants (~4.4–5.0 mg/L) exceeded the quantity required to induce a good therapeutic effect in mice (~0.9–3.0 mg/L), as previously reported by Steidler et al. (1998, 2000) [28,29]. The variations in the SE when those SPs were fused to different KRAS mimotopes suggest that there is a connection in the charge balance or the secondary structure of the mature moieties with the SP secretion functions. The potential of *L. lactis* as a vaccine-delivery host for KRAS mimotopes was further tested in the mice model.

### 2.4. Elicitation of KRAS-Specific sIgA following Oral Administration

**Figure 3:**
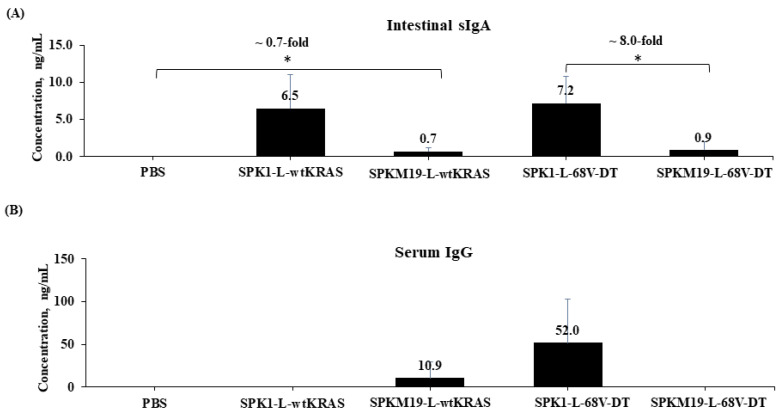
The effects of SPK1 and SPKM19 in eliciting (**A**) antigen-specific IgA from mice intestinal washes and (**B**) antigen-specific IgG from mice sera after oral immunization with recombinant *L. lactis* secreting 68V-DT KRAS and wtKRAS antigens. All values are reported as mean concentration ± standard deviation on day 22. Significant differences, *p* < 0.05, were reported between test groups and control groups (PBS), as well as between SP groups, as indicated by an asterisk (*).

The determination of mice serum IgG and intestinal secretory IgA (sIgA) antibodies was performed using indirect ELISA, as shown in Figure 3. Oral immunization with a film containing *L. lactis* secreting wtKRAS (NZ-SPKM19-L-WT) significantly induced an elevation in antigen-specific intestinal secretory IgA (sIgA) titer compared to the PBS control group (*p* < 0.05) (Figure 3A). Similarly, an elevated KRAS-specific IgA titer was observed in the immunized 68V-DT groups (*p* > 0.05). Antigen-specific serum IgG was also elevated in both KRAS-treated groups but insignificantly compared to the control group (*p* > 0.05) (Figure 3B). Comparison within treated groups to determine the effect of different SPs on Ig levels revealed the superiority of SPK1 by ~8 folds compared to SPKM19 (Figure 3A).

The ability of the SPK1 to mount a higher antigen-specific sIgA immune response than the SPKM19 after oral administration of the recombinant *L. lactis* secreting 68V-DT KRAS in mice is consistent with the previous in vitro finding, which also showed a higher capability of the SPK1 in aiding secretion of 68V-DT in *L. lactis* (Figure 3B). Therefore, it was presumed that the higher immunogenic response observed for SPK1 resulted from a higher antigen SE. This subsequently increased the amount of soluble KRAS antigens that were exposed to the gut-associated lymphoid tissue (GALT), thus promoting better accessibility to the antigen-presenting immune cells. It should also be noted that KRAS proteins that are localized and sequestered intracellularly are also able to induce immune responses but at a relatively slower and less specific rate through whole-cell endocytosis.

## 3. Discussion

In this study, *L. lactis* NZ9000 was assessed for its ability to produce two therapeutic KRAS mimotopes, 68V-DT and wild-type KRAS, using two different signal peptides, SPK1 and SPKM19, for mucosal vaccine delivery application. The 68-V mimotope (YKL**D**VVGAVGVGKS) (Ng et al., 2018) [14] is a modified mutant of KRAS G12V with an additional point mutation of valine to aspartic acid at position 7 (V7D) of the MHC groove binding site flanking the original mutation (G12V). It was previously predicted to confer immunomodulant properties in silico by promoting high affinities toward human and mouse MHC-II and -I molecules, as well as B cell epitopes. The 68-V was also shown to trigger pro-inflammatory TNF-α responses against antigen-treated PBMC in vitro and elevated specific IgG antibodies in mice sera compared to the G12V control (Ng et al., 2018) [14]. In this study, the 68-V was fused with a carrier molecule, diphtheria toxoid (DT) (QSIALSSLMVAQAIPLVGE) (Diethelm-Okita et al., 2000) [30], which served as a CD4+ T cells immune modulator to improve the antigenicity of the mimotope and the chemical stability. The diphtheria toxoid is originally based on the modified toxin produced by *Corynebacterium diphtheriae* and generally given to children over 7 years old in combination with the tetanus and pertussis vaccine (DTP). It is considered one of the safest vaccines available by the World Health Organization (WHO) (Bramwell et al., 2003) [31]. Meanwhile, the wild-type KRAS (wtKRAS) epitope (GenBank Accession Number: M54968.1), which is a gene expressed in all healthy humans, is derived from the first 50 residues linear epitope of human V-Ki-ras2 Kirsten rat sarcoma.

In order to develop an efficient live mucosal vaccine, the expression and optimized secretion system via the Sec pathway of the *L. lactis* host were utilized. Previously, we have reported the development of a novel signal peptide (SP) SPKM19, derived from SPK1, that carries mutations at the C-terminal region by incorporation of a consensus motif A-X-A-A (at position −3, −1, +1), which served as an improved binding site for SPase I for efficient recognition, cleavage, and secretion of the target protein in *L. lactis*. The optimized SPKM19 successfully improved secretion efficiency by up to 88% for the heterologous protein, *Staphylococcal* nuclease (NUC) (Alias et al., 2022) [21], and fusion of both SPs with a propeptide LEISSTCDA has further enhanced secretion (Alias et al., 2023) [32]). In this study, the optimized Sec system of *L. lactis* via the SPK1 and its derivative SPKM19 was further utilized for secretion of the therapeutic mutant 68-DT KRAS and wtKRAS proteins, as an alternative to the most commonly used lactococcal SP, USP45. Targeting the KRAS mimotopes for extracellular production is important, as it enables direct interaction between the antigen and the target mucosal environment (GIT) for the induction of a more robust and specific immune response.

In silico analysis is a useful and reliable tool to predict the performance of a signal peptide prior to the experimental settings. Here, we have initially predicted that the SPKM19-LEISS would have superior performance in aiding the secretion of the target KRAS proteins as compared to the wild-type SPK1-LEISS via the SignalP D-score values. Nevertheless, an opposite effect of the SP performance was demonstrated in *L. lactis*, with the wild-type SPK1 showing superiority in aiding the secretion of both the KRAS mimotopes, 68V-DT and wtKRAS, compared to its derivate SPKM19. The discrepancy in the performance of the SPs compared to the in silico prediction and our previous study is presumed to be due to the different properties of the MPs used, which are also reflected in the overall structure conformation of the SP and MP fusion proteins. We have shown that the performances of the SPK1 and SPKM19 vary across different proteins used (KRAS vs. NUC) and further concluded that an SP performance is highly dependent on the nature of the MP it is fused to. The propeptide LEISSTCDA is a 9-bp anionic linker that carries an overall net negative charge (−2) (Le Loir et al., 2001) [23]. In the present study, an attempt to reduce the variation in SP performances due to different fusion MPs has been initially performed by incorporating the anionic linker, LEISSTCDA, immediately downstream of the SP C-terminal domain to retain a similar net negative charge of at least the first 10 residues of the mature moieties. However, the effect of different MPs on the SP performances as indicated by the discrepancies in the final secretion yield and SE were still observed.

Le Loir et al., 2005 [33], have previously demonstrated that the nature of MPs such as size limits and structure conformation could attribute to the variation in secretion yield, with protein conformation having a more prominent effect than the protein size in impairing SE. In the present study, physicochemical analysis of the sequences on the different mature moieties revealed the NUC and KRAS proteins’ striking differences in their sizes and the net charge balance (Table 2 NUC is a ~20 kDa protein that carries an overall net global positive charge (+3), while both the KRAS peptides, 68V-DT and wtKRAS, are ~7 kDa in size and harbor an overall net global negative charge. It is suggested that the exceptionally small size of the KRAS peptide could have a more significant effect on the entire SP-MP charge balance and structure conformation, if any, as compared to when a bigger fusion protein such as NUC was used. This substantial effect on a small protein such as KRAS could make it more susceptible to impaired SE, presumably through disoriented structure conformation that impairs secretion, than for a bigger and more stable protein such as NUC.

The effect of impaired SE due to a small protein size limit has been reported in previous studies by Zhang et al. (2010) [34] that showed poor SE for a small protein, the human interferon-alpha (~10 kDa), when fused to either SP, USP45-LEISS (SE, 12%) or SPlpA-LEISS (SE, 26%). Similarly, Freitas et al. (2005) [35] showed a detrimental effect on SE for the smallest proclaimed non-bacteriocin ever secreted in *L. lactis*, a *Streptomyces tendae* antifungal protein 1 (Afp1) with ~9.8 kDa, where no detectable anti-fungal activity was observed even in a concentrated induced supernatant culture due to ineffective secretion. In *L. lactis*, despite successful translocation across the plasma membrane, improperly folded protein would not be recognized by the SPase 1 for cleavage of the SP and release of the MP to the extracytoplasmic region for secretion, and thus they would be retained in the intracellular regions. This is similarly seen in the present study, with the majority of the KRAS proteins accumulating in the intracellular fraction rather than secreted to the culture supernatant, leading to poor SE (SE approx. 25%). A new challenge in effectively secreting small peptides via the Sec system in *L. lactis* is thus highlighted in this study. Further studies to understand the localization of the target protein within the host cells during secretion and the secondary structure conformation of the SP-MP fusion proteins via computational prediction can be investigated to improve this limitation.

For vaccine delivery applications, the production of both intracellular and extracellular target antigens by the *L. lactis* host is essential. In this study, it is suggested that extracellular production of KRAS would contribute better to the robustness and specificity of the immune response triggered due to better accessibility of immune cells to the soluble KRAS for antigen presentation. On the contrary, intracellular KRAS production, where proteins are mostly retained in the host cell, would require the processing of the whole host by immune cells before the appropriate KRAS antigen can be presented on the immune cell surfaces (Rescigno et al., 2001) [36]. Mucosal delivery through oral administration using a live non-pathogenic host such as LAB is a promising vaccine delivery system with the potential of eliciting both the innate and specific adaptive immune response against mucosal diseases such as colorectal cancer (Wells and Mercenier, 2008) [16]. To induce a highly desired immune response, the bioavailability of the delivery host, the dosage of the immunogenic peptides, and the duration of exposure to the mucosal surface are essential. In this study, the property of the transient *L. lactis* was enhanced through protective double-coating of the bacteria with mucoadhesive and pH-dependent enteric-coated polymers. This has added to its value as an antigen-delivery host for mucosal vaccines.

In this study, a formulation of 4% sodium alginate and 2% Lycoat RS570, a modified polymer from pea starch, was used to develop the inner mucoadhesive film containing *L. lactis*. It was previously tested to be the best formulation that would give optimum tensile strength, elongation flexibility, and thickness to the film. The ideal film thickness was determined to be within a range of 20–30 µm (Tan et al., 2019) [37]. Sodium alginate, which is a natural polymer from brown seaweed extracts, has become one of the most widely used components for oral films due to its bioadhesive property in addition to being edible, biodegradable, non-toxic, and relatively inexpensive (Sachan et al., 2009, Hariyadi and Islam, 2020) [38,39]. Additives such as starch can be added to sodium alginate to improve its mechanical and chemical stability (Wu et al., 2001) [40]. The film-coated recombinant *L. lactis* was developed using the air convection drying method at 30 °C for 48 h, which was previously shown as a better method than the conventional spray-drying method (Fazilah et al., 2019) [41], with cell viability retainment up to 93% recovery after casting into films (Tan et al., 2019) [37]. It is suggested that the mucoadhesive property of the film would help prolong the transit time of the recombinant *L. lactis* along the lower GIT tract to promote antigen contact with the mucosal surfaces and subsequently increase uptake by the immune cells such as M cells, dendritic cells (DCs), and macrophages for priming of T cells in Peyer’s patches, or the polymeric IgA that is secreted by mature plasma cells in the lamina propria (Wells and Mercenier, 2008) [16]. The alginate is, however, susceptible to an acidic environment, resulting in crackling and loss of mechanical properties (Divya and Nampoothiri, 2015) [42]. Thus, it could benefit from protection by an outer layer.

Subsequently, the formulated mucoadhesive film containing *L. lactis* was cast with Eudragit L100-55, a degradable copolymer at a specific pH of around 5.3 (Tan et al., 2019) [37] in order to give physical protection to *L. lactis* against high acidity in the digestive tract and simultaneously enhance survivability in the high alkalinity environment of the GIT. Previously, it was shown that after 4 h exposure in the respective simulated sites, 97% of the *L. lactis* coated with Eudragit survived in pH 2 simulated gastric digestion (SGD), while 61% survivability was shown in pH 8 simulated intestinal digestion (SID). On the contrary, only 29% survivability in SGD and 58% viability in SID were shown for *L. lactis*-free cells (Tan et al., 2019) [37]. The Eudragit-coated capsule loaded with *L. lactis* film was previously demonstrated to be able to improve digestive and gastrointestinal cell survival in vitro. In this study, as the mouse has a gastric pH of 3.0 and enteric pH of 5.2 (McConnell et al., 2008) [43], it is suggested that the dissolution of the outer enteric-coated Eudragit upon reaching the GIT would expose the inner mucoadhesive film containing expressed *L. lactis* to the GALT and promote adherence along the mucosal GIT long enough to subsequently trigger the desired host immunity towards the KRAS antigens.

For antitumor responses, two types of cell responses are desired: the B cells’ humoral immune response and the T cell-mediated immune response. In peptide vaccine design, the T cell-mediated immune response is often desired (Koido et al., 2013) [44]. Interestingly, in the present study, we have shown a promising paradigm shift in the immune response to humoral immunity through antibody activation of the KRAS-specific IgA triggered following immunization with *L. lactis* secreting KRAS. The systemic antibody response is mainly of the IgG class and only to a minor extent of the IgA class. On the contrary, the mucosal antibody response is mostly of the IgA class (Aggerbeck et al., 1997) [45]. In this study, since the dominant isotype induced was IgA class rather than IgG class, it is suggested that mucosal immunity rather than systemic immunity was successfully triggered following immunization with the recombinant *L. lactis* secreting KRAS. The positive IgA response was presumed to be positively related to the oral route of immunization used in this study. One of the major benefits of using LAB as an antigen delivery host is its ability to evoke antigen-specific secretory IgA response at mucosal surfaces (Steidler et al., 1998; Qiao et al., 2021) [28,46]. Similarly, here, we showed that the engineered *L. lactis* NZ9000-secreting KRAS as an antigen delivery host could induce the desired mucosal IgA response via the oral route of immunization. However, it should be noted that the number of mice used in this preliminary study is small (n = 4); this is a limitation that should be taken into consideration when interpreting the results.

Following the in vitro assessments in mice model, it was proven that the SPK1 is superior to the SPKM19 in inducing immunogenic response against the 68V-DT, thus supporting the previous in vitro finding, which also showed the higher ability of SPK1 in targeting secretion of the 68V-DT with regard to the final yield of secreted proteins in *L. lactis*. Therefore, in this study, it was presumed that the higher immunogenic response observed for SPK1 resulted from a higher antigen secretion efficiency. This subsequently increased the amount of soluble KRAS antigens that were being exposed to the GALT, thus promoting better accessibility to antigen-presenting immune cells such as M cells, dendritic cells (DCs), and macrophages to evoke the desired mucosal immune responses. It should also be noted that KRAS proteins that are localized and sequestered intracellularly are also capable of inducing the desired immune responses, but at a relatively slower and less specific rate through whole-cell endocytosis. Based on the strong correlation between in vitro and in vivo studies, it is suggested that the quantity of secreted antigens produced could be a limiting factor in triggering the desired immune responses when utilizing *L. lactis* as the vaccine delivery host.

## 4. Materials and Methods

### 4.1. In Silico Characterization of Signal Peptide and Mature Proteins

The SPs SPK1 and SPKM19 were fused to the propeptide LEISSTCDA and denoted as SPK1-LEISS or SPKM19-LEISS. These were then fused to the mature proteins (MPs) 68V-DT and wtKRAS, yielding different SP-MP fusion genes, which were characterized for their SPs functionality via SignalP 4.1 server (https://services.healthtech.dtu.dk/services/SignalP-4.1/, accessed on 16 February 2020) (Petersen et al., 2011) [47]. The SP probability and function were measured by the *D-score* value set at a threshold of 0.4 for Gram-positive organisms, and a value higher than the threshold indicates functional SP. The SP cleavage site (at position −3 to −1) and net charge balance were also determined. Additionally, the first 10-nucleotide sequence and the net charge balance of the MP region immediately downstream of the SP cleavage site were also characterized using the SignalP 4.1 server. The physicochemical properties of the different MPs when fused with different SP genes were characterized using the ExPASy ProtParam server (http://web.expasy.org/protparam/, accessed on 16 February 2020) (Gasteiger et al., 2005) [48]. The parameters measured for the MPs region include molecular weight, theoretical pI, instability index, aliphatic index, grand average of hydropathicity (GRAVY) index and the net charge of the first 10 and 19 residues, and the global net charge balance.

### 4.2. Bacterial Strains, Plasmids, and Antibiotics

The bacterial strains and plasmids used in this study are listed in Table 3. *L. lactis* NZ9000, which was used as an expression host, was grown in M17 media (Merck, Darmstadt, Germany) supplemented with 0.5% (*w*/*v*) glucose (GM17) or SGM17 media (GM17 media supplemented with 0.5 M sucrose) at 30 °C as a standing culture. Meanwhile, *E. coli* Top 10, which was used as a cloning host, was grown in LB media (Merck, Darmstadt, Germany) at 37 °C with agitation at 250 rpm. When necessary, antibiotics, chloramphenicol, or kanamycin at concentrations of 7.5 μg/mL and 50 μg/mL, respectively, were added to the media to select for positively transformed *L. lactis* or *E. coli* bacteria.

### 4.3. PCR Amplification of SPs and KRAS Mimotopes

The list of primers used in this study is listed in Table 4. The oligonucleotides of *SPK1* and *SPKM19* fused to an anionic linker, *LEISSTCDA*, to improve secretion performance were amplified from previously constructed plasmids, pNZ-SPK1-LEISS-NUC and pNZ-SPKM19-LEISS-NUC (Alias et al., 2022) [21]. Primers 1 and 2 were used to amplify the *SPK1-LEISS* gene, while primers 1 and 3 were used to amplify the *SPKM19-LEISS* gene. Two unique RE sites, *Nco*I and *Kpn*I, were included in the primers at N- and C-terminal ends of the SPs, respectively, for directional cloning. Meanwhile, the gene-coding sequences of the *68V-DT* and *wt*KRAS were amplified from the previously constructed plasmids pNZ-USP45-68V-DTD and pNZ-USP45-wtKRAS, respectively (Hoo et al., 2020) [51]. Primers 4 and 5 were used to amplify the KRAS *68V-DT* gene, whereas primers 6 and 7 were used to amplify the *wt*KRAS gene. Two RE sites, *Kpn*I and *Sac*I, were incorporated in the primers for cloning at N- and C-terminal ends of the proteins, respectively. Additionally, a six-histidine tagged sequence (*6x-His*) was also included at the C-terminal ends upstream of the stop codon and RE sites for detection of the antigens. PCR amplification reactions used for the construction of the secretory cassettes *SP-LEISS-KRAS* were performed as previously described (Alias et al., 2022) [21].

### 4.4. Construction of Secretory Plasmids in L. lactis

The amplified genes of *SPK1-LEISSTCDA* and *SPKM19-LEISSTCDA* were ligated to the KRAS genes, *68V-DT* and *wt*, respectively, via the *Kpn*I RE site, resulting in the secretory cassette *SP-LEISS-KRAS* (as shown in Figure 1). Ligation was carried out with a T4 DNA Ligase (Thermo Fisher Scientific, Waltham, MA, USA) at 25 °C for 4 h, and ligation products were purified from gel electrophoresis. The secretory cassettes were initially cloned in *E. coli* Top 10 via the plasmid, pCR™-Blunt II-TOPO^®^ Thermo, for propagation according to the manufacturer’s protocol. Subsequently, the secretory cassettes were digested with *Nco*I/*Sac*I and directionally ligated to a linearized pNZ8048 expression plasmid before electro-transformation into the competent *L. lactis* NZ9000 according to Holo and Nes (1989) [52]. As the negative control, the empty pNZ8048 plasmid was also transformed into *L. lactis* NZ9000. The putative positive transformants were selected by growing on selective SGM17 agar media supplemented with Cm followed by 2 to 3 days of incubation at 30 °C. The correct inserts were verified by digestion with the corresponding REs or by PCR amplification with vector-specific primers (primer 8 and 9). Finally, the insert sequences were confirmed by DNA sequencing analysis.

### 4.5. Expression and Characterization of KRAS Mimotopes by Western Blotting

KRAS protein expression was performed by growing the recombinants *L. lactis* in a fresh GM17 media supplemented with 7.5 μg/mL of Cm to an OD_600nm_ = ~0.4 followed by the addition of 40 ng/mL of nisin to induce protein expression. For optimization, protein expression was continued for 2, 4, 6, or 8 h before 40 mL of the culture was harvested at each time point via centrifugation at 4000× *g* at 4 °C for 10 min. The cell pellets and supernatant media were then separated and processed accordingly. To obtain the intracellular protein fractions, the cell pellets were washed once in 1X PBS, resuspended in 1:10 of culture volume with 1X PBS, and sonicated using a 3 mm diameter probe and Omni Ruptor 400 Ultrasonic Homogenizer (Omni International, Kennesaw, GA, USA) set to 40% power, pulsed for 8 min with 30 s interval each time until the lysate became clear. Meanwhile, to obtain the extracellular protein fractions, culture supernatants were subjected to trichloroacetic acid (TCA) precipitation, as described by Koontz (2014) [53] by incubation with 1/10 culture volume of 100% (*w*/*v*) TCA (Merck, Darmstadt, Germany) on ice for 2 h, followed by centrifugation at 14,000× *g* for 10 min at 4 °C, before finally being washed twice with 1/10 culture volume of 100% (*v*/*v*) acetone. The air-dried extracellular protein pellets were finally resuspended in 1/100 culture volume of ice-cold 1X PBS. The crude intracellular and extracellular protein fractions were quantified using Bradford assay and normalized to the same concentration with 1X PBS for parallel comparison in the SDS-PAGE and Western blotting assays. SDS-PAGE assay was performed using 12% resolving gel and 4% stacking gel, while Western blotting assay for KRAS protein detection was detected using a primary antibody (1:1000 mouse anti-His.Tag^®^ Monoclonal Antibody, Novagen, Madison, WI, USA) at 4 °C overnight without agitation and secondary antibody (1:1000 horseradish peroxidase (HRP) conjugated goat anti-mouse IgG, Amresco, Cleveland, OH, USA) for another 1 h at RT with agitation as previously described (Alias et al., 2022) [21].

### 4.6. Quantification of KRAS Mimotopes by Indirect Enzyme-Linked Immunosorbent (ELISA) Assay

The yield and secretion efficiency of KRAS proteins produced by all recombinants in the intracellular and extracellular fractions were quantified by indirect ELISA using a His-Detector Western Blot HRP Colorimetric kit (KPL, Gaithersburg, MD, USA). Expression was performed at OD_600nm_ = ~0.5 with 40 ng/mL of nisin for 6 h, and the intracellular and extracellular protein fractions were harvested and processed from 40 mL culture as previously described. For the ELISA assay, a total of 100 μL of crude intracellular and extracellular protein samples were added to a 96-well Maxi Binding microtiter plate (SPL Life Sciences, Pocheon-si, South Korea) and incubated for 1 h. As a negative control, 100 μL of 1X PBS was also added to the plate. The plate was then blocked for 5 min with 200 μL of 1% (*w*/*v*) bovine serum albumin (BSA) diluted in PBST (1X PBS + 0.1% (*w*/*v*) Tween 20) before the plate was allowed to dry for 2 h. Afterward, the plate was added to 100 μL of 1:1000 HisDetector Nickel-NTA (nitrilotriacetic acid) conjugated HRP antibody and incubated for 2 h, followed by the addition of 100 μL of TMB substrate for 10 min to detect the KRAS proteins. The reaction was stopped by adding 100 μL of hydrogen peroxide, and the plate was read at 450 nm using FLUOstar^®^ Omega (BMG Labtech, Ortenberg, Germany). The normalized mean of protein concentration from three replicates was calculated using a standard curve of known concentration of BSA as the protein standard. The secretion efficiency (SE) was calculated from the ratio of secreted proteins per total proteins (sum of intracellular and extracellular fractions) produced.

### 4.7. Double Coating of Recombinants L. lactis-Secreting KRASs into Edible Films for Oral Immunization

Recombinant *L. lactis*-secreting KRASs were double-coated with mucoadhesive and enteric pH-dependent polymers, respectively, for oral delivery into the mice according to Tan et al. (2019) [37] with some modifications. Protein expression was induced with 40 ng/mL nisin at OD_600nm_ = ~0.5 to an OD_600nm_ = ~2.8 (6 h expression). A total of 70 mL of culture was then harvested by centrifugation at 14,000× *g*, 4 °C for 10 min and the cell pellet was extracted to prepare recombinant *L. lactis* film containing ~ 1.0 × 10^9^ CFU/mg. To prepare the inner coating of the *L. lactis* film, the cell pellet was washed two times with 1/10 culture volume of 1X PBS and resuspended in a final volume of 1 mL of 1X PBS. The cell suspension was mixed with 15.0 mL of inner coating base mixture containing 4% (*w*/*v*) alginic sodium salt from brown algae (Sigma-Aldrich, St. Louis, MO, USA) and 2% (*w*/*v*) Lycoat RS720 (Roquette, Lestrem, France), autoclaved at 121 °C and 15 psi for 20 min and allowed to cool at room temperature. The *L. lactis* film suspension was carefully poured into a casting tray and placed in a convection oven at 30 °C for 2 days to allow the film to dry. Dried *L. lactis* films were removed from the tray and cut into pieces weighing 1.0 mg each in order to fit into a dosing syringe for the mice feeding procedure and kept at 20 °C. Meanwhile, the enteric pH-dependent outer coating Eudragit suspension was prepared by dissolving 0.34 M methacrylic acid-ethyl acrylate copolymer (1:1) (Sigma-Aldrich, St. Louis, MO, USA), 22.6 mM triethyl citrate (Sigma-Aldrich, St. Louis, MO, USA), 69.2 mM talcum powder (Zulat Pharmacy Pte Ltd., Kuala Lumpur, Malaysia), and 62.6 mM titanium (IV) oxide (Sigma-Aldrich, St. Louis, MO, USA) in diluent water containing 50% (*w*/*v*) isopropanol and 35% (*v*/*v*) acetone in a 50.0 mL Falcon tube. The Eudragit suspension was poured into a Petri dish to set before being placed into a convection oven at 30 °C for 2 h to dry the polymer. After 2 h, the thin layer of dried Eudragit was peeled off from the Petri dish and cut into pieces, which were then used to coat each of the 1.0 mg of the recombinant *L. lactis* film. The double-coated *L. lactis* was kept at 4 °C until the commencement of mice feeding.

### 4.8. Animal, Immunization Schedule and Sample Extraction

A total of 4 female (n = 4), 6–8 weeks old BALB/c mice per group were orally immunized with a film containing recombinant *L. lactis* (~10^9^ CFU) from the strains (1) NZ-SPK1-L-68V-DT, (2) NZ-SPKM19-L-68V-DT, (3) NZ-SPK1-L-WT, and (4) NZ-SPKM19-L-WT using a size M rodent dosing syringe (Torpac Inc., Fairfield, NJ, USA). Negative control mice (n = 4) were orally fed with 200 μL of 1X PBS, pH 7.4, using a 22-gauge × 1.0-inch ball tip feeding needle of a 1.0 mL syringe. All the animal conduct, related use, and import activities of the living modified organism were performed in accordance with the approved Institutional Animal Care and Use Committees form (UPM/IACUC/AUP-R038/2017) of Universiti Putra Malaysia, Serdang, Malaysia BALB/c mice were purchased from the Animal Resources UnitUPM, Serdang, Malaysia, and housed at the Laboratory of Vaccines and Immunotherapeutic (LIVES) at the Comparative Medicine and Technology UnitUPM, Serdang, Malaysia. The treated and negative control mice groups were primarily immunized on Day 1, followed by two booster immunizations on Day 8 and Day 15.

Mice were euthanized on Day 22 by cervical dislocation (Carbone et al., 2012) [54], and samples were collected. Prior to the euthanization, all mice were anesthetized intraperitoneally with 20 µL of a Ketamine (93.3 mg/kg)/Xylazine (1.33 mg/kg) cocktail (Vetoquinol UK Ltd., Towcester Northamptonshire, UK) per 20.0 g of mice body weight, according to Parasuraman et al. (2010) [55]. Mice lower GI tracts of mice, including small and large intestines, were harvested to obtain intestinal wash samples. The intestinal tracts were cleaned with 1X PBS before being dissected open, and the inner surfaces were then flushed with 2.0 mL of 1X PBS solution, pH 7.4, containing 1.0 mM PMSF protease inhibitor (Sigma-Aldrich, St. Louis, MO, USA). The washed solution was allowed to stand for 10 min before being centrifuged at 3000× *g* at 4 °C for 10 min, after which the top layer supernatant containing intestinal washes was separated and stored at −20 °C. The extracted whole blood was collected in collection tubes containing anticoagulant K_2_EDTA (BD, Franklin Lakes, NJ, USA) and stored at −20 °C. Sera were collected from whole blood that was allowed to clot for 60 min on ice, followed by centrifugation at 3000× *g* at 4 °C for 10 min, before the top layer containing serum was obtained and stored at −20 °C.

### 4.9. Immunoglobulins (sIgA and IgG) Detection by Indirect ELISA

For IgA and IgG detection, 100 µL of undiluted mice intestinal wash samples from a small intestine and large intestine (1:1) or 50 µL of diluted mice sera (1:10), respectively, were used for indirect ELISA assay as previously described (Siak et al., 2021) [56]. A volume of 100 µL of 5 µg/mL of each synthetic peptide (68-V and wild-type KRAS) was used for initial coating on microtiter plates, followed by blocking with 0.1% (*w*/*v*) Tween 20 and BSA (1–2%, *w*/*v* at 4 °C overnight incubation with either the samples or standard (1X PBS)) for 1 h at RT. A volume of 100 µL of 1:2000 HRP conjugated goat anti-mouse IgA polyclonal antibody (Invitrogen, Waltham, MA, USA) or 1:7500 HRP-conjugated goat anti-mouse IgG polyclonal antibody (Santa Cruz Biotechnology, Dallas, TX, USA) was added for 1 h at RT for the detection of bound IgA/IgG. Finally, 100 µL of TMB substrate was added for 5 min, and the reaction was stopped with 100 µL of 2N H_2_SO_4_. The plate was read using FLUOstar^®^ Omega (BMG Labtech, Ortenberg, Germany) at a detection wavelength of 450 nm and a reference wavelength of 570 nm. The concentrations of both IgA and IgG were calculated based on their respective standard curves.

### 4.10. Statistical Analysis

All experimental data were reported as a relative mean (n = 3) ± standard deviation. Data were subjected to statistical analysis using Student’s paired *t*-test with a significance threshold value at *p* < 0.05.

## 5. Conclusions

The present study is the first that reported the use of SPK1 as an alternative to USP45 in vaccine delivery application using the GRAS *L. lactis* host. Conclusively, the SP SPK1 is shown to be superior to SPKM19 for the secretion of KRAS peptides in *L. lactis.* The ability of the *L. lactis* in mediating successful secretion and elevated humoral immunity through KRAS-specific sIgA production represents a possibility for this vaccine host to be used as the delivery vehicle for future mucosal vaccine developments especially targeting metastatic colorectal cancer. Nevertheless, this study is considered preliminary, and future studies to validate the anti-cancer prophylactic and therapeutic efficacy of the live recombinant *L. lactis* secreting KRAS can be further validated in pre-clinical disease challenge studies with a significantly larger sample size.

## Figures and Tables

**Table 1:** In silico signal peptide prediction of the fusion proteins.

Fusion Genes	SP Length (aa)	D-Score	SPase I Cleavage Site (−3 to −1)	Net Charge of SP	First 10 Residues of MP (+1 to +10)	Net Charge of First 10 Residues of MP
SPK1-LEISS-68V-DT	23	0.710	VHA	+3	LEISSTCDAG	−1
SPKM19-LEISS-68V-DT	23	0.815	AHA	+2	ALEISSTCDA	−1
SPK1-LEISS-wtKRAS	23	0.711	VHA	+3	LEISSTCDAG	−1
SPKM19-LEISS-wtKRAS	23	0.808	AHA	+2	ALEISSTCDA	−1

The in silico analysis of the predicted signal peptide (SP) region, SPK1-LEISS or SPKM19-LEISS, and mature protein (MP) region, 68V-DT KRAS or wild-type KRAS, was performed via SignalP 4.0 server. The SP probability, D-score, of 0.4 was used as a threshold, where a value similar to or higher than the threshold indicates a functional SP. The SP length and net charge and also the cleavage site of signal peptidase (Spase I) at the C-terminal of SP from position −3 to −1 are shown. The amino acid sequence and net charge of the first 10 residues of the MPs immediately downstream of the SPase I cleavage site, starting at position +1 to +10, are indicated.

**Table 2:** Physicochemical analysis of the different mature protein moieties fused with different signal peptides.

Fusion Genes	MP Length	MP Size (kDa)	Charge of First 10 MP	Charge of First 18 MP	MP Total Charge	MP pI	MP Gravy Index	MP Aliphatic Index	MP Instability Index
SPK1-L-68V-DT	61	6.53	−1	−2	−2	6.65	−0.02	95.90	21.24
SPKM19-L-68V-DT	62	6.59	−1	−2	−2	6.66	0.01	95.97	21.06
SPK1-L-wtKRAS	67	7.43	−1	−2	−6	5.46	−0.35	87.16	25.97
SPKM19-L-wtKRAS	68	7.50	−1	−2	−6	5.46	−0.32	87.35	25.73
SPK1-L-NUC	186	20.80	−2	−3	+3	8.78	−0.86	65.06	33.77
SPKM19-L-NUC	187	20.87	−2	−3	+3	8.78	−0.85	65.24	33.64

The amino acid (aa) length, size, charge of the first 10 and 18 residues, isoelectric point (pI), GRAVY, aliphatic index, instability index, and overall net charge of the mature proteins (MP) were analyzed using the ExPASy ProtParam server.

**Table 3:** Bacterial strains and plasmids used in this study.

Bacterial Strains and Plasmids	Relevant Features	Reference
Strains		
*E. coli* TOP 10	Propagation host for pCR™-Blunt II-TOPO^®^	Invitrogen, Waltham, MA, USA
*L. lactis* NZ9000	Expression host for nisin-inducible plasmid pNZ8048	De Ruyter et al. (1996) [49]
Plasmids		
pCR™-Blunt II-TOPO^®^	*E. coli* cloning plasmid containing *pUC* ori, *Kn^r^*, *lacZα* genes; 3.5 kb	Invitrogen, Waltham, MA, USA
pNZ8048	*L. lactis* nisin inducible expression plasmid; containing *P_nisA_* promoter and *Cm^r^*; 3.3 kb	Kuipers et al. (1998) [50]
pNZ-SPKM19-LEISS-wtKRAS	pNZ8048 harboring *SP_SPKM19_-LEISS-wild-type KRAS* cassettes with C-terminal six-histidine tag; 4.0 kb	This study
pNZ-SPK1-LEISS-wtKRAS	pNZ8048 carrying *SP_SPK1_-LEISS-wild-type KRAS* cassettes with C-terminal six-histidine tag; 3.97 kb	This study
pNZ-SPKM19-LEISS-68V-DT	pNZ8048 carrying *SP_SPKM19_-LEISS-68V-DT* cassettes with C-terminal six-histidine tag; 3.92 kb	This study
pNZ-SPK1-LEISS-68V-DT	pNZ8048 carrying *SP_SPK1_-LEISS-68V-DT* cassettes with C-terminal six histidine tag; 3.98 kb	This study

**Table 4:** List of primers used in this study.

Gene (SP-MP)/Plasmid	Primer	Sequence from 5′ to 3′	T_a_ (°C)	Amplicon Size (bp)
***SPKM19-LEISS***	(1) F-Spk1	CCATGGCTATGAAAAAAATATTAAC	47	119
	(2) R-M19LEISS	GGTACCTGCATCACAAGTCGACGATATTTCGAGAGCATGTACTG		
***SPK1-LEISS***	(1) F-Spk1	CCATGGCTATGAAAAAAATATTAAC	47	110
	(3) R-Spk1LEISS	GGTACCTGCATCACAAGTCGACGATATTTCGAGAGCATGTACATTC		
**KRAS *68V-DTD***	(4) F-68V-DT	GGTACCATGTATAAATTAGATGTTGTTG	48	165
	(5) R-68V-DT	GAGCTCCTAATGATGATGATGATGATGT		
**KRAS** wild-type (***wt*KRAS**)	(6) F-wtKRAS	GGTACCATGACTGAATATAAACTTGTGGTAGTT	52	183
	(7) R-wt*KRAS*	GAGCTCCTAATGATGATGATGATGATGG		
***SPKM19-LEISS-wt*** **KRAS**	(1) F-Spk1	CCATGGCTATGAAAAAAATATTAAC	53	292
	(7) R-wt*KRAS*	GAGCTCCTAATGATGATGATGATGATGG		
***SPK1-LEISS-wt*** **KRAS**	(1) F-Spk1	CCATGGCTATGAAAAAAATATTAAC	53	289
	(7) R-wt*KRAS*	GAGCTCCTAATGATGATGATGATGATGG		
***SPKM19-LEISS-68V-DT***	(1) F-Spk1	CCATGGCTATGAAAAAAATATTAAC	46	277
	(5) R-68V-DT	GAGCTCCTAATGATGATGATGATGATGT		
***SPK1-LEISS-68V-DT***	(1) F-Spk1	CCATGGCTATGAAAAAAATATTAAC	51	274
	(5) R-68V-DT	GAGCTCCTAATGATGATGATGATGATGT		
pNZ8048	(8) F-pNZ8048	TATTGTCGATAACGCGAGCA	55	Varies with insert size
	(9) R-pNZ8048	CGTTTCAAGCCTTGGTTTTC		

## Supplementary Materials

The following supporting information can be downloaded at https://www.mdpi.com/article/10.3390/ijms24108928/s1.
