# Supplementary material for: Effect of Secretion Efficiency of Mutant KRAS Neoantigen by Lactococcus lactis on the Immune Response of a Mucosal Vaccine Delivery Vehicle Targeting Colorectal Cancer
Source: Int J Mol Sci. 2023 May 18;24(10):8928. doi: 10.3390/ijms24108928 (PMC10219268; doi:10.3390/ijms24108928)
Supplement: Supplementary file 1 [file ijms-24-08928-s001.zip › ijms-2348261-supplementary.pdf]

Supplementary Data

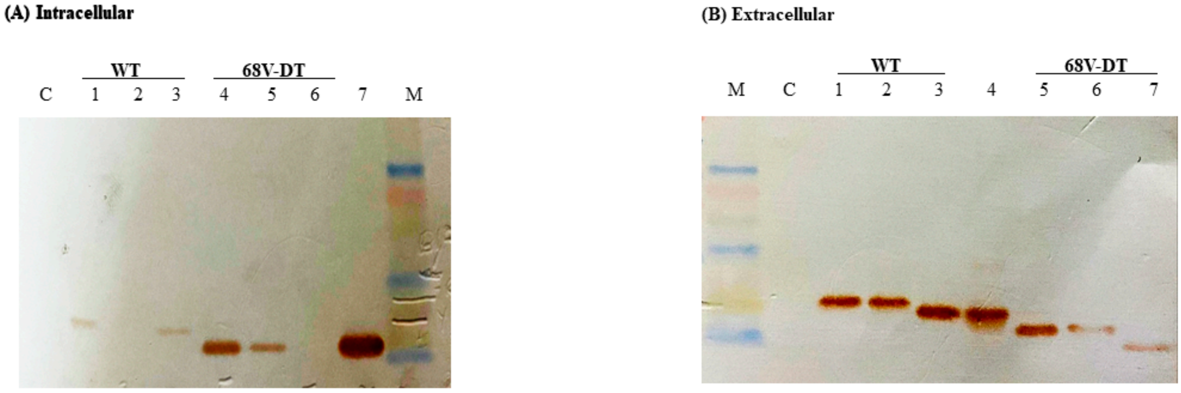

Scheme 1: Expression and secretion analysis of wtKRAS and 68V-DT by the recombinants *L. lactis* with 40 ng/ml of nisin at 6 h. Figure (A) showing the intracellular protein fraction. M, Rainbow™ Protein Ladder; C, NZ9000 with empty vector pNZ8048 (negative control). Lane 1: NZ-SPKM19-L-WT; Lane 2, NZ-USP45-WT; Lane 3: NZ-SPK1-L-WT; Lane 4: NZ-SPKM19-L-68VDT; Lane 5: NZ-SPK1-L-68VDT; Lane 6: NZ-USP45-68VDT; Lane 7: positive control (NZ-Xyl). Figure (B) showing the extracellular protein fraction. Lane 1: NZ-SPKM19-L-WT; Lane 2: NZ-SPK1-L-WT; Lane 3: NZ-USP45-WT; Lane 4: positive control (NZ-Xyl); Lane 5: NZ-SPKM19-L-68VDT; Lane 6: NZ-SPK1-L-68VDT; Lane 7: NZ-USP45-68VDT. The positive control (NZ-Xyl) was included only for the Western blotting analysis. The NZ-USP45-WT and NZ-USP45-68VDT were not used in this study, thus were not further reported.
